# Key insights into cannabis‐cancer pathobiology and genotoxicity

**DOI:** 10.1111/adb.70003

**Published:** 2024-11-13

**Authors:** Albert Stuart Reece, Gary Kenneth Hulse

**Affiliations:** ^1^ University of Western Australia Crawley Western Australia Australia; ^2^ School of Health Sciences Edith Cowan University Joondalup Western Australia Australia

**Keywords:** cannabis, chromothripsis, micronucleus

## Abstract

Whilst mitochondrial inhibition and micronuclear fragmentation are well established features of the cannabis literature mitochondrial stress and dysfunction has recently been shown to be a powerful and direct driver of micronucleus formation and chromosomal breakage by multiple mechanisms. In turn genotoxic damage can be expected to be expressed as increased rates of cancer, congenital anomalies and aging; pathologies which are increasingly observed in modern continent‐wide studies. Whilst cannabinoid genotoxicity has long been essentially overlooked it may in fact be all around us through the rapid induction of aging of eggs, sperm, zygotes, foetus and adult organisms with many lines of evidence demonstrating transgenerational impacts. Indeed this multigenerational dimension of cannabinoid genotoxicity reframes the discussion of cannabis legalization within the absolute imperative to protect the genomic and epigenomic integrity of multiple generations to come.

Recent papers in Science provide penetrating and far‐reaching insights into the mechanisms underlying micronuclear rupture a key genotoxic engine identified in many highly malignant tumours.^1^, ^2^ Reactive oxygen species (ROS) generated either by damaged mitochondria or the hypoxic tumour microenvironment were shown to damage micronuclear envelopes, which made them more sensitive to membrane rupture. Damage occurred by both increased susceptibility to membrane rupture and impaired membrane repair. Micronuclear rupture is known to be associated with downstream chromosomal shattering, pan‐genome genetic disruption by chromothripsis, widespread epigenetic dysregulation and cellular ageing. Clinical expressions of genotoxicity are expected to appear as cancer, birth defects and ageing.

CHMP7 (charge multivesicular body protein 7) oxidation caused heterodimerization by disulphide crosslinking and aberrant cross‐linking with membrane bound LEMD2 (LEM‐domain nuclear envelope protein 2) inducing membrane deformation and collapse. ROS‐CHMP7 directly induced chromosomal shattering. Oxidized CHMP7 bound covalently to the membrane repair scaffolding protein ESCRT‐III (endosomal sorting complex required for transport–III). ROS triggered homo‐oligomerization of the autophagic receptor p62/sequestome re‐routing the CMPH7‐ESCRT‐III complex away from membrane repair into macroautophagy via the autophagosome and microautophagy via lysozomes.^1^, ^2^, ^3^ Expected downstream consequences of micronuclear rupture including chromosomal fragmentation, chromothripsis and cGAS‐STING (cyclic adenosine‐guanosine synthase–stimulator of interferon signalling) activation were demonstrated. Cancer‐related innate inflammation is known to drive tumour progression and distant metastasis. These principles were tested both in normal and also numerous malignant (including head and neck squamous, cervical, gastric, ovarian and colorectal cancers) cell lines.^1^, ^2^ Similar processes including DNA damage and epigenomic derangements have also been identified in T_H_1‐lymphocytes during fever indicating that mitochondriopathic‐genotoxic mechanisms may in fact be widespread and fundamental.^4^

Cannabis has been known to be linked with both micronuclear development and mitochondrial inhibition for many decades.^5^, ^6^

All cannabinoids have been implicated in genotoxicity as the moiety identified as damaging the genetic material is the central olivetol nucleus on the C‐ring itself.^7^ This finding implicates Δ8‐, Δ9‐, Δ10‐, Δ11‐tetrahydrocannabinol, cannabigerol, cannabidiol and cannabinol amongst all other cannabinoids.

Historically, the cancer‐cannabis link has been controversial. Differing results in published studies may be attributed to various factors including multiple exposures (including tobacco), differences in study design and the rapid rise of cannabis potency. One often quoted study actually specifically excluded high level cannabis exposure, which would now appear to have been a major methodological limitation.^8^ It is widely documented that there has been a sharp increase in cannabis concentration from the 1970s to the present day. THC concentrations of 25%–30% are commonly noted in cannabis herb and flower sold commercially, and 100% THC concentrations are well known for cannabinoid based products such as dabs, waxes and ‘shatter’.

In this context, the recent appearance of a series of continent‐wide epidemiological, space–time and causal inferential studies in both Europe and North America is notable for many positive signals for various cancers including breast, pancreas, liver, AML, thyroid, testis, lymphoma, head and neck squamous cancer, total childhood cancer and childhood ALL.^9^, ^10^, ^11^, ^12^, ^13^, ^14^, ^15^ The literature on cannabis and testicular cancer is almost uniformly positive and has a relative risk of around 2.6‐fold,^16^ this risk factor is now widely acknowledged^17^, ^18^, ^19^ and the effect is quite fast since the median age of exposure may be about 20 years and the median age of testis cancer incidence is only 31 years. Testicular cancer is the adult cancer responsible for the most years of life lost.^17^, ^18^, ^20^, ^21^ The inclusion of several childhood cancers in association with cannabis exposure obviously implicates transgenerational transmission of malignant mutagenesis.

An intriguing finding in the case report literature is that in many cases, cancers occur decades earlier and are very aggressive at diagnosis.^22^ Mechanisms such as the synergistic mitochondriopathic–micronuclear axis presently proposed in the recent Science papers^1^, ^2^, ^3^, ^4^ may directly explain this very worrying observation.

Whilst cancer is thought to be a rare outcome amongst cannabis exposed individuals, ageing effects are not. A dramatic acceleration of cellular epigenetic age by 30% at just 30 years was recently reported^23^ with indications this effect likely rises with age,^24^ and the demonstration that cannabis exposed patients had adverse outcomes across a wide range of physical and mental health outcomes including myocardial infarction and emergency room presentations.^25^ Importantly, the ageing process itself has been shown to be due to redistribution of the epigenetic machinery in such a manner as to produce dysregulation (and widespread reduction) of gene expression and to be inducible by limited genetic damage resulting from just a handful of DNA breaks.^26^ Extremely worryingly, age‐related morphological changes have been described in both oocytes and sperm.^27^, ^28^

Epidemiological studies of European and American cannabis‐cancer links are supported by epidemiological, space–time and causal inferential studies of links between cannabis and congenital anomalies.^29^, ^30^, ^31^, ^32^, ^33^ Reported congenital anomalies are clustered in the cardiovascular, neurological, limb, chromosomal, urogenital and gastrointestinal systems. The fact that all five chromosomal anomalies studied here are represented in this list, notwithstanding their high rate of known foetal loss, is strong evidence for chromosomal mi‐segregation during germ cell meiosis, which is the genetic precursor to micronucleus development.^34^, ^35^ The fact that almost identical results were reported in both the United States and Europe provides strong external validation to these findings.^30^

This is consistent with recent press reports of dramatic increases in babies and calves born without limbs in both France and Germany^36^, ^37^ raising the public health spectre of downstream implications of food chain contamination. Melbourne, Australia, is a multi‐ethnic city, which heads the global leaderboard for babies born with the serious limb anomalies amelia and phocomelia.^37^, ^38^, ^39^, ^40^ This pattern of elevated rates of major birth defects is not seen in the host nations from which these migrant populations are derived. Cannabis farms are increasingly common around Melbourne, just as they are in the French province of Ain, which has similar concerns.^37^, ^41^, ^42^, ^43^

Major epigenetic changes have been found in human sperm,^44^ which have also been identified in exposed rodent offspring.^44^, ^45^, ^46^ Indeed, 21 of the 31 congenital anomalies described following prenatal thalidomide exposure have also been observed epidemiologically following prenatal cannabis exposure and 12 of 13 cellular pathways by which thalidomide operates have been similarly identified in the cannabis mechanistic literature.^47^ Both human and rodent epigenomic studies^44^, ^45^, ^46^ and epidemiological studies show that adult cannabis exposure is linked with the incidence of autism^48^, ^49^, ^50^, ^51^, ^52^, ^53^ and cerebral processing difficulties^54^, ^55^, ^56^, ^57^ in children prenatally exposed. Together, this data is clear and robust evidence for the transgenerational transmission of major genotoxic outcomes.

Notwithstanding the well‐known ambiguities in the epidemiological literature for cannabis, it is clear from the above brief overview that there is strong and compelling evidence that cannabis genotoxic outcomes are well substantiated and form a remarkably congruent skein of interrelated evidence across all three domains of genotoxic pathology including cancer, congenital anomalies and ageing.

So too compelling epidemiological, morphological and epigenetic evidence of transgenerational transmission of cannabinoid genotoxicity to foetus, egg, sperm and offspring carries far reaching and transformative implications and indeed reframes the discussion surrounding cannabis legalization from merely personal‐hedonistic to the protection of the national genomic integrity for multiple subsequent generations.

The present time therefore represents a watershed moment. The new profoundly insightful studies from Science point the way and provide the trigger. Clearly, there is a great need for a new and updated cohort of epidemiological studies on these issues at the population level in the modern context of the widespread availability of much more potent cannabinoid preparations.

However, our first responsibility is to act on the evidence we do have. Given the uniform picture painted by data from myriad directions, it can be said that the evidence for cannabinoid genotoxicity is at once so clinically significant, robust and compelling as to constitute a resounding clarion call to action: The only outstanding question is ‘Will we rise to the challenge?’

## CONFLICT OF INTEREST STATEMENT

The authors declare no conflicts of interest.
